# Supplementary material for: Content-rich biological network constructed by mining PubMed abstracts
Source: BMC Bioinformatics. 2004 Oct 8;5:147. doi: 10.1186/1471-2105-5-147 (PMC528731; doi:10.1186/1471-2105-5-147)
Supplement: Additional File 2 — The original results of the above study (non-essential files are deleted to keep the file size under the limit set by BMC bioinformatics). [file 1471-2105-5-147-S2.bz2 › chilibotAdditionalFile2/dip05/483ID8702798E10574/html/IL8RB_PPBP.html]

 


 **IL8RB** and **PPBP** 
  
Found 28 abstracts in PubMed, retrieved 05.  
 

 What does Google say? 
 PDF only 
| .edu only 

---

**Interactive relationship** (e.g. stimulation, inhibition, etc)

**Inhibitory relationship**- After phagocytosis of opsonized yeast, the expression of CXCR1 and CXCR2  [ **IL8RB** ]  was substantially down regulated and was accompanied by reduced Ca responses to corresponding ligands, IL 8 and neutrophil activating peptide 2 NAP 2  [ **PPBP** ] .  Ref: 12239185 Blood, 2002
**Neutral relationship**- The migration activating concentrations of 50 ng mL GCP 2 and NAP 2  [ **PPBP** ]  induced less potent migratory responses and FAK phosphorylation in CXCR2  [ **IL8RB** ]  expressing cells as compared with IL 8.  Ref: 12627953 Biochemistry, 2003
- CXCR1 and CXCR2  [ **IL8RB** ]  mediate migratory activities in response to IL 8 and other ELR CXC chemokines e.g., GCP 2 and NAP 2  [ **PPBP** ] .  Ref: 12627953 Biochemistry, 2003
- CXCR2  [ **IL8RB** ]  binds IL 8 and other structurally related chemokines such as growth related oncogene GRO a, GRObeta, GROgamma, neutrophil activating peptide 2 NAP 2  [ **PPBP** ]  and epithelial cell derived neutrophil activating peptide 78 ENA 78.  Ref: 12365735 Fish Shellfish Immunol, 2002

**Non-interactive relationship** (e.g. studied together, co-existance, homology, etc.)

- Transendothelial migration of neutrophils in response to CXCL7  [ **PPBP** ]  or IL 8 CXCL8 was subject to modulation by CTAP III, but not CXCL4, as seen by drastic desensitization of the migratory response of neutrophils pre exposed to CTAP III, which was paralleled by selective down modulation of CXCR 2  [ **IL8RB** ] .  Ref: 12193731 J Immunol, 2002
- This was evident from experiments in which inhibition of the CTAP III processing protease and simultaneous blockade of the CXCL7  [ **PPBP** ]  high affinity receptor CXCR 2  [ **IL8RB** ]  led to complete abrogation of CTAP III mediated neutrophil adhesion.  Ref: 12193731 J Immunol, 2002
- Consistent with this conclusion is inhibition of the chemotactic response to monocytes by agents that block CXCR2  [ **IL8RB** ] , the receptor that NAP 2  [ **PPBP** ]  uses.  Ref: 12101278 J Leukoc Biol, 2002
